# Air Pollution and Endurance Exercise: A Systematic Review of the Potential Effects on Cardiopulmonary Health

**DOI:** 10.3390/life15040595

**Published:** 2025-04-03

**Authors:** Sofía González-Rojas, Rodrigo Yáñez-Sepúlveda, Marcelo Tuesta, Braulio Sánchez-Ureña, José Trejos-Montoya, Jorge Olivares-Arancibia, José Francisco López-Gil, Daniel Rojas-Valverde

**Affiliations:** 1 Centro de Investigación y Diagnóstico en Salud y Deporte (CIDISAD-NARS), Clínica de Lesiones Deportivas (Rehab&Readapt), Escuela Ciencias del Movimiento Humano y Calidad Vida (CIEMHCAVI), Universidad Nacional de Costa Rica, Heredia 86-3000, Costa Rica; sofia.gonzalez.rojas@est.una.ac.cr (S.G.-R.); braulio.sanchez.urena@una.ac.cr (B.S.-U.); jose.trejos.montoya@una.ac.cr (J.T.-M.); drojasv@hotmail.com (D.R.-V.); 2 Faculty Education and Social Sciences, Universidad Andres Bello, Viña del Mar 2520000, Chile; rodrigo.yanez.s@unab.cl; 3 Exercise and Rehabilitation Sciences Laboratory, School of Physical Therapy, Faculty of Rehabilitation Sciences, Universidad Andres Bello, Viña del Mar 2520000, Chile; marcelo.tuesta@unab.cl; 4 Laboratory of Sport Sciences, Centro de Medicina Deportiva Sports MD, Viña del Mar 2521156, Chile; 5 AFySE Group, Research in Physical Activity and School Health, School of Physical Education, Faculty of Education, Universidad de las Américas, Santiago 7500000, Chile; jolivares@udla.cl; 6 One Health Research Group, Universidad de Las Américas, Quito 170124, Ecuador

**Keywords:** physical activity, cardiovascular, VO_2max_, environmental, contamination

## Abstract

This systematic review aimed to analyze the implications of endurance exercise in environments with certain levels of air pollution. This study was developed on the basis of the consensus of the Preferred Reporting Items for Systematic Reviews and Meta-Analyses (PRISMA). The present review is supported by articles containing the main databases PubMed, Elsevier, and Web of Science (WoS), including scientific articles published in the last 20 years. This study highlights that exposure to air pollution during endurance activities, such as cycling and outdoor running, significantly affects cardiopulmonary health. In conclusion, while physical exercise in environments with high air pollution presents significant risks to cardiopulmonary health, implementing preventive measures and adopting public policies are crucial to minimizing these impacts and promoting safe exercise practices. Likewise, on the basis of these results, it is possible to motivate the creation of safe and natural spaces for sports practice.

## 1. Introduction

Air pollution is a major environmental concern, particularly in urban areas, where the burning of fossil fuels from transportation, agriculture, and industry significantly contributes to the presence of airborne contaminants. These pollutants include suspended particulate matter (PM_2.5_), nitrogen oxides (NO_2_), carbon dioxide (CO_2_), ozone (O_3_), among others [1]. Additionally, natural air purification processes have been compromised by deforestation and urban living habits, such as poor waste management and the widespread use of single-use materials [2]. As a result, climate change has worsened, further influencing the dispersion and transport of pollutants, which interact with factors such as humidity and temperature to affect air quality [3].

The health consequences of air pollution are well documented, extending beyond respiratory conditions (e.g., chronic obstructive pulmonary disease, lung cancer, asthma) [4,5,6,7] to include cardiovascular [8], renal [9], immune [10], gastrointestinal [11], metabolic [12], and mental health effects [13]. Studies have established a correlation between air pollution exposure and mortality rates, independent of age, sex, race, smoking habits, alcohol consumption, diet, and obesity [14]. Furthermore, the COVID-19 pandemic has exacerbated the mortality risks associated with long-term exposure to air pollutants [15,16,17,18]. Regular physical exercise, particularly endurance activities such as prolonged cycling or running, is widely recognized for its health benefits. However, performing endurance exercise in polluted environments introduces potential health risks, which are not fully understood [19]. Exposure to high concentrations of PM_2.5_ during physical activity may reduce its expected health benefits, limit adherence to exercise programs, and increase physiological stress [20]. Specifically, inhaling particulate matter and other pollutants can increase blood pressure, impair vascular function, and increase oxidative stress and inflammation [21]. These effects were linked to metabolic disturbances, particularly in the arginine and proline pathways, which play crucial roles in cardiorespiratory and metabolic health [22,23]. While many studies suggest negative health impacts of exercising in polluted environments, some research challenges this assumption. For example, studies on urban cyclists have reported no acute effects on neuroplasticity, inflammation, or performance [19,24]. Similarly, in competitive endurance sports such as triathlons, exposure to O_3_ and PM_2.5_ was observed to affect performance, yet professional athletes seem less sensitive to these changes [25]. Furthermore, evidence suggests that long-term exposure to air pollution can contribute to declines in cardiovascular function [26,27] and reductions in physical performance, particularly among female athletes [28]. Despite the growing body of research, there is a lack of consensus regarding the true extent of the risks and benefits of endurance exercise in polluted environments. While some studies highlight detrimental effects, others suggest that adaptations to long-term exposure or differences in individual susceptibility may play a crucial role in mitigating health risks. This study is distinguished by its originality and critical approach, offering a comprehensive analysis of the interplay between aerobic exercise and air pollution—a topic of increasing relevance in public health and sports science.

Unlike previous studies that have examined these factors in isolation, this review compares the benefits of endurance exercise with the potential health risks posed by air pollution. By synthesizing recent studies, this review aims to identify inconsistencies in the literature and highlight gaps in knowledge regarding the dose–response relationship between air pollution and exercise performance; analyze the methodologies, biomarkers, and participant characteristics used in existing research to assess how different populations are affected; and provide evidence-based recommendations for minimizing the risks associated with endurance exercise in polluted environments, such as choosing optimal training times and locations. Ultimately, this research aims to inform health professionals, athletes, and policymakers about the health implications of endurance exercise in polluted environments, especially in urban settings [29]. For this reason, this systematic review aims to analyze the implications of endurance exercise in environments with certain levels of air pollution. Similarly, this research could help professionals raise awareness of the health implications of endurance exercise in polluted environments, especially in urban areas.

## 2. Materials and Methods

### 2.1. Search Strategy

This study was developed on the basis of the consensus on Preferred Reporting Items for Systematic Reviews and Meta-Analyses (PRISMA) [30]. The present review is supported by a set of articles containing the main databases PubMed, Elsevier, and Web of Science (WoS), including scientific articles published in the last 20 years. The keywords used for this review are air pollution OR air pollution AND endurance OR cycling OR running OR triathlon (search performed in English). They were used to search and select published data. In addition, the articles to be selected met the following inclusion and exclusion criteria (Table 1): a. original articles on the topic; b. available in the abovementioned databases; and c. years of publication up to August 2024. The present systematic review did not consider the use of gray literature.

### 2.2. Information Search and Extraction Strategy

For the development of the research question, the PICO strategy (patient or problem, intervention, comparison, and outcome or results) was used.

Considering the selection criteria, two groups of researchers (S.G.-R., B.S.-U., J.T.-M., and D.R.-V.; and R.Y.-S., J.O.-A., and J.F.L.-G.) independently performed an internal review. In the case of discrepancies in the selection of articles, a third investigator (J.F.L.-G.) resolved the differences, deciding on the inclusion or exclusion of studies as appropriate.

### 2.3. Study Selection: Inclusion and Exclusion Criteria

Original scientific articles published between 2004 and August 2024 were included if they evaluated the effects of endurance exercise (e.g., cycling, running, or triathlon) performed under air pollution exposure. Eligible studies were required to be in English, involve human participants aged 18–65 years, and include pre- and postexercise assessments of air pollution-related variables (e.g., PM_2.5_, O_3_, NO_2_, and CO_2_). Studies also had to evaluate cardiovascular health indicators such as resting heart rate, stroke volume, or spirometry. Only full text articles were considered, and all types of physically active populations were included, regardless of training status. Studies were excluded if they were incomplete, had low scientific evidence (e.g., opinion pieces, editorials), involved populations with noncardiovascular noncommunicable diseases, included nonhuman subjects, were unpublished (preprints), were not citable, or focused on activities not classified as endurance exercise (e.g., motor cycling, public transportation, walking, or other mobility-related activities) (Table 1).

### 2.4. The Characteristics of the Studies

Studies that met the selection criteria were published between 2004 and 2024. A total of 14 studies were published between 2004 and 2014, and 10 were reported between 2015 and 2024. A review of studies evaluated the effects of air pollution on cardiovascular and respiratory health during moderate intensity physical exercise, most of which involved various endurance exercises, such as cycling and outdoor running. In addition, the studies were conducted at different latitudes, with Canada, the United States, and the Netherlands standing out, as well as other countries such as Australia, Brazil, Belgium, Colombia, Scotland, England, Sweden, and Turkey.

### 2.5. Characteristics of Participants/Interventions

A total of 559 participants included in the 24 articles of the present review were considered, of which 253 were men, representing 45%, and 109 were women, representing 20%. In addition, we considered a set of studies with a sample of 197 participants with no sex distribution, representing 35% of the studies. On the other hand, the distributions by sex and cycling modality included in the studies corresponded to *n* = 139, 65% men, and *n* = 75, 35% women. Similarly, in the running modality, the number of runners included corresponds to *n* = 67, 78% men, and *n* = 18, 22% women. The predominant exercise intensity was moderate (*n* = 21, 75%), followed by moderate to high (*n* = 6, 21%), and submaximal (*n* = 1, 4%). All the participants were amateur and recreational in both running and cycling. The selected investigations included a variety of specifications on the basis of the level of practice (e.g., amateur and recreational), category (e.g., noncyclists, cyclists, nonrunners, runners, runners, according to intensity (e.g., low and medium intensity) modality (e.g., running, cycling), physical condition (e.g., physically active, sedentary), and health condition (e.g., healthy).

### 2.6. Study Design

The types of studies and designs varied among the studies that were included (see Table 2), and there was a greater concurrence of crossover study design, accounting for 46% of the studies (7 of 24), which used it to compare the effects of high and low exposure to air pollution. Additionally, 36 percent (10 studies) employed an experimental design with controlled exposure to pollutants such as DE (diesel), and the remaining 18% (5 studies) were comparative. Most studies included both males and females, with a range of participants from *n* = 7 to *n* = 53.

### 2.7. Air Pollution Variables and Cardiopulmonary Health Variables

In this review, the effects of air pollution on health during endurance physical activities were analyzed, and the following environmental pollution variables were identified: PM_2.5_ (42%), PM_10_ (27%), NO_2_ (19%), O_3_ (15%), DE or diesel exhaust (19%), BC or black carbon (12%), PM_0.1_ (4%), NO (4%), NO_X_ (4%), CO (4%), PM_1.0_ (4%), NO_3_ (4%), and total particles (4%).

In this review, we analyze the effects of air pollution on cardiovascular health during the practice of endurance physical activities. The following variables were evaluated: heart rate (HR) 14%, heart rate variability (HRV) 11%, systolic blood pressure (SBP) 11% (DBP) 11%, and energy consumption (VCO_2_) 1%. Endothelial function (FMD) 4%, respiratory function (FEV_1_, FVC, PEF, PEF, PEF, PFP, FEF25-75) 15%, and residual volume (RV) 4% were also evaluated. Other inflammatory markers, such as IL-6, IL-10, and cytokines, were measured in 11% of the patients; bronchial compliance in 4%; nasal resistance in 4%; and other variables, such as oxygen saturation (SPO_2_), maximal oxygen consumption (VO_2max_), and tidal volume (VT), in 15%.

## 3. Results

**Figure 1:**
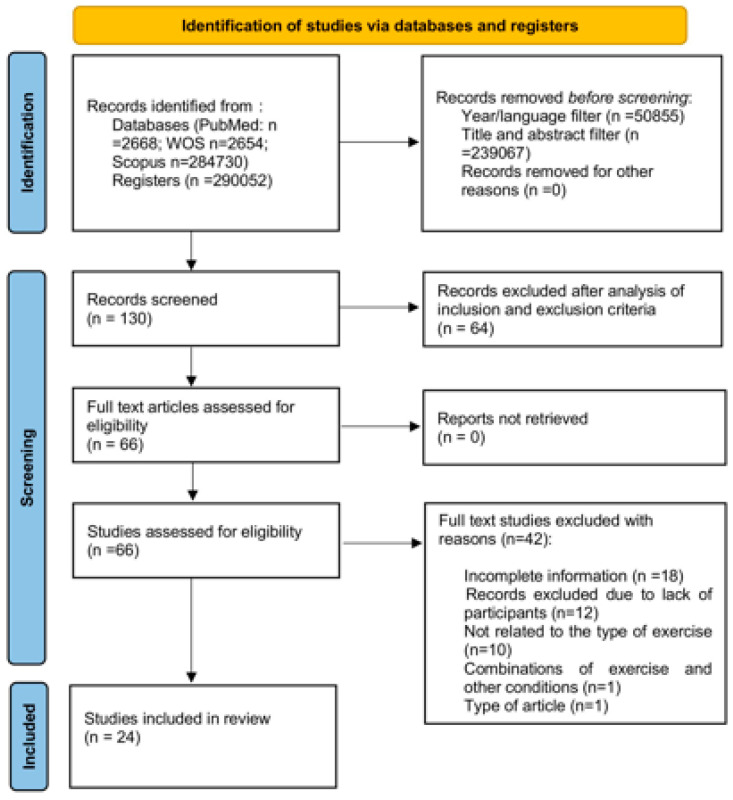
PRISMA 2020 flow diagram.

Figure 1 shows the general results of the systematic review process, where author, country, year, and population characteristics, among other particularities of each study, were analyzed.

### Results of Studies

We included 12 studies focused on evaluating the impact of exercise in air pollution on cardiovascular adaptations, of which only one reported an increase in the exercise HR after chronic cycling (3 days) at moderate intensity [49] but without changes in the resting HR [39,41]. Similarly, no change in resting heart rate was observed with acute running at moderate intensity [52]. In addition, two studies reported a decrease in resting heart rate variability [32,33], and three reported impaired vascular function [33,46] after chronic exercise (cycling) in a polluted environment, but another reported no difference [39,43]. The latter was also observed as an acute effect of cycling in response to pollutant exposure [51]. With respect to blood pressure, two studies reported an increase in SBP and DBP during chronic (3 days) [33] and acute (after) [38] cycling, whereas one reported no effect after chronic exposure [43]. Considering blood biomarkers of cardiovascular risk, such as interleukins (e.g., serum IL-1, IL-6) and endothelin, no changes were observed after chronic exposure to moderate intensity, short-duration exercise for seven days in a polluted environment [53]. However, some biomarkers are altered by acute exposure to pollution. In this sense, an increase in blood IL-6 (and VEGF) was reported after 90 min of pedaling at moderate intensity in a polluted environment [35]. In contrast, no changes were observed after 5.7 km at the same exercise intensity [40].

In relation to lung function, 10 studies reported no effects or increases in lung function [31,44,48]; however, another three studies reported that pollution exposure can induce a decrease in lung function (i.e., CFV, FEV, and/or PEFR and FEF [36,37,45]). With respect to lung inflammation, an increase in the levels of adhesion bronchial molecules such as sP-selectin, sE-selectin, sICAM-1, sVCAM-1, IL-6, and/or VEFG (i.e., pulmonary inflammation) was observed. For example, after prolonged (>60 min) light [34], moderate cycling [35,45] was observed. However, another study did not show changes in sP-selectin, sE-selectin, sICAM-1, or sVCAM-1 after seven days of cycling from 30 min per day to moderate intensity exposure to exhaust gases [42]. Additionally, other biomarkers related to pulmonary inflammation, such as fractionated exhaled nitric oxide (FeNO), increased after cycling for 120 min for four days [36]. However, other studies either reported no change after 30 min of moderate intensity pedaling over 6 days [44] or even reported a decrease (also of nitrate in condensed exhaled breath) after 30 min of high intensity running [54]. With respect to cellular effects, cyclists who cycled 5 to 10 km for 7 days at moderate intensity in a polluted environment presented an increase in airway macrophage carbon compared with noncyclists [53].

As shown in Table 2, the duration of exposure to exercise during cycling or running in a polluted environment varied considerably. Acute exposure duration ranged from 15 to 90 min. Chronically, participants were exposed to urban environments for a minimum of 2 [47,48,54] to a maximum of 16 days [31]. Sample sizes were relatively small, with most studies including both men and women accounting for 11 of the investigations [31,32,34,36,37,38,39,40,41,45,48,53], whereas only 1 study focused exclusively on females [33] and another 12 focused exclusively on males [35,42,43,44,45,46,47,49,50,51,52,54].

Eleven studies evaluated exposure to fine particles of particulate matter (PM_2.5_), and seven evaluated exposure to coarse particles (PM_10_) as the main environmental variable. In addition to fine and ultrafine (PM_2.5_) [32,33,35,36,37,38,45,47] and (PM_1_ and PM_0.1_) [32,40,54] particulate matter, exposure to high levels of ultrafine particles and gases such as NO_2_ and NOx [35,36,37,38,40,45,47,54] has a direct effect on cardiovascular and/or pulmonary health during physical activity. Within this group, five studies also considered exposure to O_3_, highlighting their adverse effects on lung function during physical activity [33,36,45,47,52]. In addition, eight studies assessed exposure to fumes from fuels such as diesel [34,39,42,43,44,46,49,50], but the results are controversial. Here, only five studies reported adverse effects on lung function during exercise [34,43,46,49,50].

## 4. Discussion

A systematic review revealed that exposure to air pollution during endurance activities, such as cycling and outdoor running, significantly affects cardiopulmonary health [32,33,34,35,36]. The results revealed modifications at the inflammatory and pulmonary levels in the athletes. The included studies revealed that common pollutants from vehicular traffic, such as particulate matter (e.g., PM_2.5_, PM_10_), NO_2_, and O_3_, are associated with inflammatory responses and reduced lung function [35,36,37,46,49,54]. Specifically, changes in heart rate (HR) [32,39,43,49], blood pressure (BP) [33,35,37,39,46], and forced vital capacity (FVC) [33,36,45,54] can occur following exposure to these pollutants, as detrimental effects can occur even after brief exposures of only 60 min [34,35,45].

The studies reviewed show great diversity in terms of the populations that were evaluated, the type of exercise, and the conditions to which the subjects were exposed. Most of the investigations focused on urban environments [31,41,49,50,51,52,53,54], where pollution levels are relatively high, and crossover designs have been used to compare the effects of exposure to high and low concentrations of pollutants from vehicular traffic and other pollutants already in the environment, such as O_3_, NOx, and NO_2_ [32,33,36,37,47,54]. Although some studies have reported no significant acute effects [31,40,41,50,54], the general trend indicates that prolonged or repeated exposure to high levels of pollution can exacerbate the negative effects on cardiovascular and respiratory health [35,37,46,50].

In contrast to the findings of previous studies, these results are consistent with existing evidence that air pollution increases oxidative stress and inflammation [55,56,57]. This may reduce the benefits of physical exercise but not limit them completely. For example, studies have shown that exposure to polluted air is associated with decreased physical performance and adherence to physical activity, which is consistent with the results of this systematic review [58,59,60,61]. Furthermore, variability in potential risks and physiological responses to contamination may be influenced by factors such as age [59,62,63] and sex [64,65,66], which highlights the importance of conducting more specific studies exploring these variables.

In other instances, exercising in places with high air pollution could impair cardiopulmonary health via various mechanisms, and air pollution affects cardiovascular health through multiple interconnected mechanisms. The inhalation of PM_2.5_, NO_2_, O_3_, and CO generates oxidative stress, increasing the level of reactive oxygen species (ROS) and reducing the level of antioxidants, which damages endothelial cells and promotes systemic inflammation through the release of IL-6, TNF-α, and IL-1β. This causes endothelial dysfunction, decreases nitric oxide (NO), and promotes vasoconstriction. In addition, activation of the sympathetic nervous system increases blood pressure and alters heart rate variability, increasing the risk of arrhythmias. Chronic exposure also induces a procoagulant state, increasing platelet aggregation and thrombus formation, which can lead to hypertension, atherosclerosis, heart attack, and stroke. These effects, in addition to increasing cardiovascular mortality, compromise the body’s ability to benefit from aerobic exercise in polluted environments. Exposure to air pollutants [67,68,69], such as fine particulate matter [45,70,71,72,73,74] and vehicle-emitted gases [33,75], can induce oxidative stress and inflammation, affecting vascular and respiratory function.

This not only compromises the proper functioning of the cardiopulmonary system during exercise but also increases the risk of developing chronic diseases, such as hypertension and chronic obstructive pulmonary disease (COPD) [36,76,77].

In addition, these gases generate physiological reactions at the endothelial and platelet levels, increasing the tendency for thrombus formation [78,79]. Impairment of the endothelium, which is the layer lining the inside of blood vessels, is especially important because it interferes with normal vessel function, decreases the ability of vessels to dilate, and promotes clot formation [80,81,82]. Additionally, prolonged exposure to these pollutants triggers chronic low-grade systemic inflammation. These changes contribute to an increase in blood pressure and an increased predisposition to adverse cardiovascular events [81,83,84]. In summary, exposure to air pollutants affects the cardiovascular system through a sequence of events, including impaired endothelial function, increased clot-forming capacity, and intensified inflammation, all of which contribute to impaired cardiovascular health. Despite this, it is important to consider that a study that examined whether exercising indoors versus outdoors reduced the cardiorespiratory effects of outdoor air pollution in older adults revealed that polluted outdoor exercise increased cardiovascular stress and malondialdehyde, leading to decreases in heart rate variability, demonstrating that exercise in a polluted environment negatively affects the cardiovascular response compared with indoor exercise [85].

These findings highlight the importance of addressing air pollution reduction issues to protect cardiovascular function and prevent related diseases. Additionally, some of the included studies revealed that although physical activity is generally beneficial to health, these adverse effects may offset the benefits, especially in areas with high levels of pollution [35,36,37,39].

From a public health perspective, a considerable challenge is faced in attempting to balance the promotion of physical activity with the need to reduce exposure to environmental pollutants [86,87,88]. Some strategies to address this problem include encouraging indoor exercise with air filtration systems [89,90,91,92], engaging in physical activity during off-peak vehicular traffic hours [93,94,95], and establishing policies that reduce air pollution levels, such as establishing and expanding low emission zones in urban areas to limit vehicle traffic that does not meet certain environmental standards; establishing air quality monitoring networks to provide accurate and real-time information on pollution levels; implementing warning systems to inform the public about high-pollution episodes; and supporting research on the health effects of air pollution and the effectiveness of mitigation policies [96,97,98]. It is recommended that people identify elevated levels of contamination by reviewing specialized pages, as contaminants are varied and can change from place to place.

It is very relevant to consider that exposure in controlled protocols is different from what occurs in the real world, since most of the studies conducted in controlled environments have shown negative results, whereas studies conducted in real contexts with exposure to normal city traffic have shown more positive results. This can be explained by considering that in a controlled environment, one seeks to isolate variables to study the effect of a specific contaminant. However, in real life, organisms are exposed to a mixture of chemical, physical, and biological agents, in addition to factors such as stress, diet, and climatic fluctuations. This mixed and continuous exposure can trigger epigenetic responses that modulate gene expression in a more complex and cumulative manner. That is, while in the laboratory, the effect of a single agent is studied under constant conditions, in the environment, a network of modifications (such as DNA methylation and histone modifications) is activated that can enhance or dampen the response to contamination [99].

These findings emphasize the urgent need for policy interventions and urban planning strategies to mitigate the impact of air pollution on physically active populations. Policymakers should prioritize low-emission zones in urban areas, enforce stricter vehicle emission regulations, and expand real-time air quality monitoring networks to provide accurate pollution data for residents. Additionally, the integration of warning systems to alert the public about high-pollution episodes can help athletes and recreational exercisers make informed decisions about outdoor training [96,97,98].

From a healthcare perspective, professionals should incorporate air quality considerations into exercise recommendations, particularly for vulnerable populations, including individuals with pre-existing cardiovascular or respiratory conditions. Health education programs should promote awareness of the risks of exercising in polluted environments and advise preventive measures, such as monitoring pollution levels through specialized websites, modifying exercise schedules to off-peak traffic hours [93,94,95], and encouraging the use of air filtration systems in indoor training spaces [89,90,91,92].

For athletes and coaches, personalized strategies should be implemented to minimize exposure to harmful pollutants. These include choosing training routes in low-traffic areas, selecting times of the day with better air quality, and incorporating alternative training methods indoors on high-pollution days. Furthermore, adaptive strategies, such as the use of particulate-filtering masks, should be explored to determine their effectiveness in reducing the inhalation of pollutants while maintaining performance. Although the evidence reviewed suggests that exposure to air pollution during endurance exercise can have adverse effects on cardiopulmonary health, this relationship is not uniform across studies. Although several papers have reported systemic inflammation, endothelial dysfunction, and reduced lung function [32,33,35,36,37,46,49,54], 61% of the studies reviewed on lung function reported no significant negative effects, suggesting that the response to pollution may depend on multiple factors, such as exercise intensity and duration, the physical condition of the individual, and adaptive mechanisms to chronic exposure. This finding highlights the need for nuanced interpretation and future research exploring possible mechanisms of physiological resilience and the factors that modulate individual susceptibility to environmental pollution during exercise. Although the burden attributable to air pollution has decreased in recent years, it remains a significant public health risk. Current estimates provide a solid foundation to guide effective health interventions. In this context, there is an urgent need to accelerate the transition to cleaner household energy sources in low-resource communities, with the aims of reducing health risks, promoting sustainable development, and supporting physical activity in healthier environments [100].

**Figure 2:**
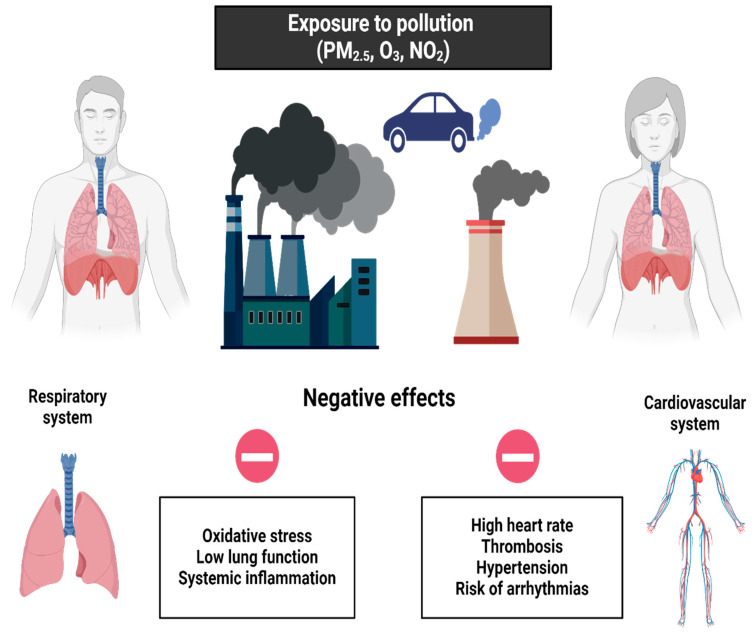
Impact of exercise in air-polluted environments on cardiovascular and pulmonary health.

While physical exercise in environments with high air pollution presents significant risks to cardiopulmonary health (summarized in Figure 2), the implementation of preventive measures and the adaptation of public policies are crucial to minimize these impacts and promote safe exercise practices. Likewise, on the basis of these results, it is possible to motivate the creation of safe and natural spaces for sports practice.

**Figure 3:**
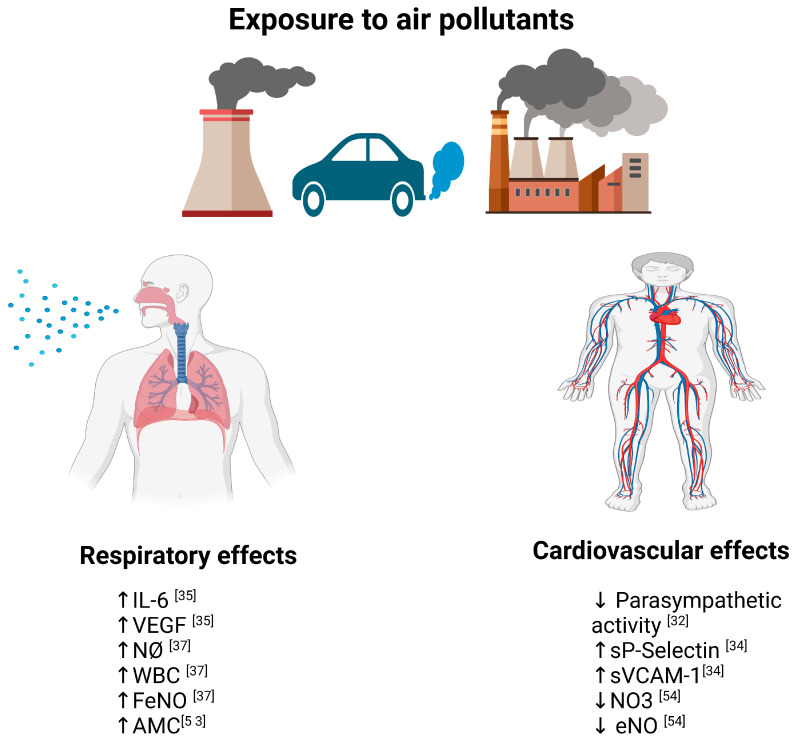
Potential mechanisms responsible for cardiopulmonary health.

Figure 3 shows the possible molecular mechanisms that explain the effects of pollution on cardiovascular health.

### 4.1. Practical Applications

The findings of this systematic review can guide the implementation of specific practices to minimize the adverse effects of exercise in environments with high air pollution. Health professionals and athletic trainers are advised to counsel their patients and athletes to perform physical activities in areas with less exposure to pollutants, such as parks away from heavily trafficked roads or sports facilities with air filtration systems. In addition, exercise should be scheduled during times of lower pollution, such as early in the morning or late at night, when pollution levels are usually lower. The timing of pollution peaks in cities should be considered when physical activity promotion programs are implemented. Other factors, such as environmental temperature and safety, should also be considered to better organize schedules for physical exercise. Monitoring air quality through mobile applications or specialized websites to avoid exercising outdoors on days with high-pollution alerts is also recommended. Finally, promoting the use of specific particulate-filtering masks can be an additional measure for those who cannot avoid physical activity in polluted areas.

### 4.2. Limitations and Future Lines of Research

The main limitations of this review include the heterogeneity of the studies in terms of design, type of exercise, exercise intensity and duration, exposure levels, and participant characteristics, which complicates the comparison of results. Many studies have small sample sizes and do not adequately control factors such as exercise intensity and adaptation to contamination. In addition, most research has focused on short-term exposures, limiting the applicability of long-term results. There is also potential publication bias, as studies with nonsignificant results may not have been reported with the same frequency.

Future research should focus on longitudinal studies that evaluate the long-term effects of exercise in environments with different levels of air pollution to better understand the chronic implications for cardiopulmonary health. It is essential to explore the efficacy of interventions such as the use of filtering face masks, modification of exercise intensity, and selection of low-pollution locations to mitigate adverse effects. In addition, studies that include a greater diversity of populations, including different age groups, people with pre-existing conditions, and different levels of adaptation to exercise, are needed to identify particularly vulnerable subgroups. It would also be valuable to investigate the interaction between air pollution and other environmental factors, such as temperature and humidity, to develop more comprehensive and accurate recommendations for safe exercise in urban environments. Despite the growing interest in the effects of aerobic exercise in polluted environments, a systematic review has identified several gaps in the literature that limit a complete understanding of the associated risks and benefits. Lack of longitudinal studies: Most research examines only acute effects without assessing the cumulative impact on long-term health. Underrepresentation of vulnerable populations: Mainly young and healthy adults have been studied, leaving out children, older adults, and people with pre-existing diseases. Inconsistencies in the measurement of pollution: Differences in the pollutants evaluated and lack of control for climatic factors limit comparisons between studies. There is little evidence on mitigation strategies: There is no consensus on which measures (e.g., use of masks, choice of schedules) are most effective in reducing the negative effects. Lack of knowledge on body adaptation: More research is needed on whether athletes can develop physiological responses that minimize the impacts of pollution.

## 5. Conclusions

According to this systematic review, endurance exercise in environments with high air pollution can have significant negative impacts on cardiopulmonary health. These include increased levels of inflammation and oxidative stress, as well as decreased respiratory function. Although physical exercise has general benefits, exposure to vehicular traffic pollutants such as fine particulate matter and gases may negate these positive effects, especially in urban areas. It is crucial to consider air quality as a key element in optimizing the positive effects of exercise and reducing potential health hazards.

To reduce these effects, measures such as physical activity in less polluted areas, choosing times of the day with less traffic, and promoting policies that seek to reduce air pollution are suggested. It is also important to continue conducting research with larger and more rigorous studies to analyze the long-term impacts of physical activity in highly polluted environments, in addition to evaluating measures that can safeguard the health of athletes and the general public. On the basis of these findings, urban design should consider monitoring environmental pollution through biosensors located at different points. The design of health policies that consider aspects such as time of day, temperature, vehicular traffic, and exposure to pollutants for the promotion of physical activity in the population is also suggested.

## Figures and Tables

**Table 1:** Eligibility criteria for studies.

Inclusion
Original scientific articles Studies on endurance exercise (e.g., cycling, running, triathlon) and performed under air pollution English language publications Include population between the age of 18 to 65 years old Include that the study contains pre and post assessment of air pollution related variables (PM_2.5_, O_3_, NO_2_, CO_2_, or other air pollution-related variables) Articles assessing cardiovascular health (resting heart rate, systolic volume, spirometry, or other) Research available in full text Publications from 2004 to date (August 2024) Include articles that include assessments of physiological changes Include all types of populations that engage in physical activity for health
Exclusion
Papers that are incomplete or low-evidence types of articles (e.g., opinion, editorials) Studies involving people with NCDs not related to cardiovascular health Research that has not yet been published (preprints) Studies that cannot be referenced Studies that have not been conducted on humans Studies involving mobility activities such as motorcycling, bus, subway, walking, or other nonresistance exercise

**Table 2:** Compilation of scientific articles.

Authors/ Year	City/ Country	Aim	Type/Design Study	Participants	Protocol	Air Pollution Markers	Cardiorespiratory Markers	Major Findings
Strak et al., 2010 [31]	Utrecht, Netherlands	To compare lung function after cycling in low vs. high vehicular traffic	Comparative study	12 healthy adults ♂ and ♀	Cycling for 60 min × 16 d at moderate intensity	PM_10_	FEV1, FVC, PEF	Elevated [PM_10_] from traffic does not affect respiratory function either immediately or 6 h after cycling
Weichenthal et al., 2011 [32]	Ottawa (ON), Canada	To compare the effects of exercise in low and high levels of vehicular traffic on HRV	Comparative and crossover study	42 healthy adults ♂ and ♀	Cycling for 60 min at moderate intensity (chronic exposure)	PM_0,1_ PM_2.5_, BC	HRV	Short-term exposure to high traffic ↓ parasympathetic activity 3 h pos-cycling
Weichenthal et al., 2014 [33]	Montreal (QC), Canada	To compare effects of exercise in low and high levels of vehicular traffic on BP, HRV, and MVF	Comparative and crossover study	53 healthy adults ♀	Cycling for 120 min × 3 d at moderate intensity	PM_2.5_, NO_2_, O_3_	BP, HRV, MVF	Short-term (2 h) to traffic pollution during physical activity affects cardiovascular function (↑ SBP and DBP, ↓ HRV, ↓ MVF) in healthy young women
Sehlstedt et al., 2010 [34]	Wellington, Australia	To investigate the inflammatory effects of diesel exhaust exposure during exercise on airway inflammation	Experimental comparative study	15 healthy adults ♂ and ♀	15 min intervals of cycloergometry during one-hour for two days at light to moderate intensity inside an exposure chamber	DE	Bronchial adhesion molecules (sP-Selectin, sICAM-1, sVCAM-1)	A higher bronchial concentration of sP-Selectin and sVCAM-1 were observed after cycling, without poststudy effects
Pasqua et al., 2020 [35]	São Paulo, Brazil	To investigate the urban air pollution on inflammatory and cardiorespiratory responses during exercise	Experimental design	10 healthy young people ♂	Cycling for 90 min at moderate intensity in both conditions, filtered and polluted air	PM_2.5_, PMT, NO, NO_2_	BP, inflammatory serum cytokines (IL-6, VEGF)	Cycling in atmospheric pollution elicits inflammatory responses (↑ IL-6, ↑ VEGF) and ↑ BP
Park et al., 2017 [36]	Sacramento (CA), USA	To compare the exposure to high and low vehicular emissions during cycling	Comparative and crossover study	32 healthy adults ♂ and ♀ (urban cyclists)	Cycling at moderate intensity for high (12 km) and low (10 km) vehicular traffic route	PM_2.5_, NO_X_, NO_2_, NO, O_3_	FVC, FEV1	>levels of UFPM (<0.1 μm) are associated with ↓ lung function (FVC and FEV1)
Kubesch et al., 2015a [37]	Barcelona, Spain	To investigate how short-term exposure to traffic-related air pollution affects respiratory and inflammatory responses	Crossover study	28 healthy adults ♂ and ♀	Cycling for 120 min × 4 d at moderate intensity in air polluted by vehicle traffic	BC, NO, PM_10_, PM_2.5_	FeNO, NØ, WBC, FEV1, FVC, FEF	Cycling in polluted air induces systemic (↑ NØ, WBC) and airway inflammatory processes (↑ FeNO), but increases lung function (↑ FEV1, ↑ FVC, ↑ FEF)
Kubesch et al., 2015b [38]	Barcelona, Spain	To compare blood pressure when cycling in low and high pollution from vehicular traffic	Crossover study	28 healthy adults ♂ and ♀	Cycling for 120 min × 1 d at moderate intensity in air polluted by vehicle traffic	BC, NO, PM_10_, PM_2.5_	SBP, DBP	Cycling in polluted air ↑ SBP and ↑ DBP
Koch et al., 2020 [39]	Vancouver (BC), Canada	To assess the vascular response to physical activity after β2-agonist use while breathing diesel exhaust in individuals with EIB	Experimental design	18 healthy adults ♂ and ♀	Cycling for 30 min × 4 d at moderate intensity in air polluted by DE	DE	FMD, BP, HR, CRVE, CRAE	Inhalation of diesel fumes does not significantly compromise micro and macro vascular function
Jacobs et al., 2010 [40]	Antwerp, Belgium	To observe changes in biomarkers of lung inflammation in high level vehicular traffic	Experimental design	38 healthy adults ♂ and ♀	Cycling during 5.7 km × 1 d at moderate intensity with high vehicular traffic	NO, PM_1.0_, PM_2.5_, PM_10_	IL-6, platelet function, serum protein levels, WBC	Biomarkers did not change after physical activity
Hernández et al., 2021 [41]	Bogota, Colombia	To evaluate the cyclist’s exposure to particle-related air pollution on selected bicycle lanes in Bogota	Experimental design	10 ♂ and ♀	Cycling for 30 min × 6 d to moderate intensity with high vehicular traffic	PM_2.5_, BC	VR, HR, SpO2, RR	There was no increase in both oxygen demand and inhaled air volume was identified
Giles et al., 2019 [42]	Vancouver (BC), Canada	To determine the effects of DE exposure during exercise of different intensities on adhesion molecules and inflammatory cells	Experimental design	18 adults ♂	Cycling for 30 min × 7 d at moderate intensity in high and low DE exposure	DE	sP-Selectin, sE-Selectin, sICAM-1, sVCAM-1, WBC	There was no change in blood adhesion protein or WBC
Giles, Tebbutt et al., 2018 [43]	Vancouver (BC), Canada	To determine the effects of high vs. low intensity cycling due to DE exposure	Experimental design	18 adults ♂	Low and high intensity cycling for 30 min × 7 d during exposure to high and low concentrations of DE	DE	Endothelin-1, NOx, FMD, BP	There was no change in endothelin-1, plasma NOx, FMD, or BP after exercise
Giles, Carlsten et al., 2018 [44]	Vancouver (BC), Canada	To determine the effects on pulmonary function and inflammation of high vs. low intensity cycling due to exposure to DE	Experimental design	18 adults ♂	Cycling for 30 min × 6 d at high and moderate intensity during exposure to filtered air vs. DE	DE	PEFR, FeNO, NE	There was no difference in lung function (PEFR) and inflammation (FeNO and NE) by exercise in DE vs. filtered air
Elliott and Loomis, 2021 [45]	Nevada (NV), USA	To assess lung function after exposure to fine particulate matter during exercise	Pilot study	31 urban cyclists ♂ and ♀	Cycling for 60 min × 7 d at moderate intensity and exposure to fine airborne particles	PM_10_, PM_2.5_, NO, O_3_, CO	FEV1, FVC, PEFR, MMEFR	Airborne particulate matter is associated with short-term decreases in lung function (↓ FEV1, ↓ FVC, ↓ PEFR)
Barath et al., 2010 [46]	Umeå, Sweden	To evaluate the vascular effects of diesel exhaust exposure	Crossover study	18 healthy adults ♂	Outdoor running for 120 min × 5 d of moderate intensity with exposure to particulate pollutants	DE, PM_2.5_, PM_10_	FBF, tPA	Exposure to diesel exhaust in urban areas can significantly impair vascular function
Aydın et al., 2014 [47]	Istanbul, Turkey	To evaluate nasal functions due to exposure to vehicular traffic	Crossover study	20 healthy adults ♂	Outdoor running for 60 min × 2 d at moderate intensity in high vs. low vehicular traffic	PM_2.5_, PM_10_, NO_2_, O_3_, CO	Nasal functions	Experienced more nasal irritation, congestion, and respiratory problems in polluted air than in cleaner air
Jarjour et al., 2013 [48]	Berkeley (CA), USA	To assess lung function due to exposure to fine particulate matter from high vehicular traffic	Experimental design	15 healthy adults ♂ and ♀	Cycling for 8 to 9.5 km × 2 d at moderate intensity in a high vs. low vehicular traffic environment	PM total, CO	FEV1, FVC	High exposure to pollutants may not acutely affect healthy cyclists (↔ FEV1, ↔ FVC)
Giles et al., 2012 [49]	Vancouver (VC), Canada	To determine the effect of pre-exercise exposure to DE on pulmonary function and cardio-respiratory variables during exercise	Experimental design	8 endurance-trained ♂	20 km cycling at moderate intensity pos-exposure to filtered air vs. DE for 60 min × 3 days	DE	FEV1, SpO_2_, CVF, HR_mean_	Prior exposure to DE reduced the exercise-induced bronchodilation effect and increased average heart rate during exercise
Giles et al., 2014 [50]	Vancouver (VC), Canada	To evaluate the respiratory and metabolic effects of diesel exposure in high and low intensity cycling	Experimental design	18 recreationally active ♂	Low intensity cycling (30% VO_2peak_) vs. high intensity (60% VO_2peak_) for 30 min × 6 d with exposure to DE or filtered air	DE	VE, VO_2_, VCO_2_, O_2_ cost of exercise	Low intensity cycling with DE exposure presented higher VE, VO_2_, VCO_2_, and O_2_ cost than filtered air
Cutrufello et al., 2011 [51]	Pennsylvania (PA),USA	To examine the effect of newly generated whole exhaust gases on exercise performance	Randomized crossover study	16 collegiate athletes ♂	20 min plus 6 min to moderate and heavy intensity cycloergometry, respectively, with exposure to high and low PM concentrations	PM	PP, FMD	↓ FMD and ↑ PP with ↓ of exercise performance
Gomes et al., 2010 [52]	Colinton, Scotland	To investigate the impact on lung function of exposure to a hot, humid, O3-polluted environment	Randomized crossover study	10 athletes ♂	Outdoor running of high intensity with exposure to clean environment vs. high O_3_ levels for 8 km	O_3_	HR_mean_, expired volume, VO_2_	↓ expired volume and ↓ VO_2_ during exercise in warm environment plus O_3_
Nwokoro et al., 2012 [53]	London, United Kingdom	To compare the impact on pulmonary inflammation and AMC due to exposure to polluted environment between cyclists and noncyclists	Experimental study	14 cyclists ♂ and ♀ 14 noncyclists ♂ and ♀	Cycling of 5–10 km × 7 d at moderate intensity with exposure to environmental pollution from road traffic between cyclists vs. noncyclists	BC	Serum IL-1b, IL-2, IL-6, IL-8, TNF-a, GM-CSF, AMC	Cyclists had more AMC in airway macrophages than noncyclists
Rundell et al., 2008 [54]	Pennsylvania (CA), USA	To investigate the impact on lung function, oxidative stress, and pulmonary inflammation after a race with exposure to a polluted versus a clean environment	Comparative study	12 physically fit adults ♂	Outdoor running 30 min × 2 d at high intensity (85–90% HR_max_) in high ambient vs. low vehicle traffic	PM_1_, NO_3_, NO	FEV1, FEF25-75, FeNO, NO_3_ (EBC), MDA	↓ FEV1, ↓ FEF25-75, ↓NO_3_, and ↓ eNO after exercise in high PM_1_

AMC: airway macrophage carbon; BC: black carbon; BP: blood pressure; CRAE: central retinal arteriolar equivalents; CRVE: central retinal venular equivalents; DBP: systolic blood pressure; DE: diesel exhaust; EBC: exhaled breath condensated; EIB: exercise-induced bronchoconstriction; FBF: forearm blood flow; FEF: forced expiratory flow; FeNO: fractionated exhaled nitric oxide; FEV_1_: forced expiratory volume in one second; FMD: flow-mediated vasodilation; FVC: forced vital capacity; GM-CSF: granulocyte–macrophage colony-stimulating factor; HR: heart rate; HR_mean_: mean heart rate; HR_max_: maximum heart rate; HRV: heart rate variability; IL-xx: interleukins (1b, 2, 6, 8); MDA: malondialdehyde; MVF: microvasculature function; NE: norepinephrine; NØ: neutrophil cells; NO: nitric oxide; NO_2_: nitrogen dioxide; NO_3_: nitrate; NOx: nitrogen oxides; O_3_: ozone; PEF: peak expiratory flow; PEFR: peak expiratory flow rate; PMxx: particulate matter; UFPM: ultrafine particulate matter.

## Data Availability

The material for this article is available from the corresponding author.

## Abbreviations

Abbreviations	Definition
PM_2.5_/PM_10_/PM_1_/PM_0.1_	Particulate matter with diameter ≤ 2.5 μm/10 μm/1 μm/0.1 μm
NO_2_	Nitrogen dioxide
NO/NOx	Nitric oxide/nitrogen oxides
CO	Carbon monoxide
CO_2_	Carbon dioxide
O_3_	Ozone
DE	Diesel exhaust
BC	Black carbon
UFPM	Ultrafine particulate matter
HR	Heart rate
HRV	Heart rate variability
SBP/DBP	Systolic/diastolic blood pressure
VO_2_/VO_2_max/VO_2_peak	Oxygen Uptake/Maximal Oxygen Uptake/Peak Oxygen Uptake
VCO_2_	Carbon Dioxide Output
VT	Tidal volume
VE	Minute Ventilation
FVC	Forced vital capacity
FEV1	Forced expiratory volume in 1 second
PEF/PEFR	Peak expiratory flow/peak expiratory flow rate
FEF_25–75_	Forced expiratory flow at 25–75% of the pulmonary volume
RV	Residual volume
SpO_2_	Peripheral Oxygen Saturation
FeNO/eNO	Fraction of Exhaled Nitric Oxide
NO_3−_	Nitrate
IL-xx	Interleukins (e.g., IL-1β, IL-6, IL-8)
VEGF	Vascular Endothelial Growth Factor
TNF-α	Tumor Necrosis Factor Alpha
GM-CSF	Granulocyte–macrophage colony-stimulating factor
NE	Norepinephrine
MVF/FMD	Microvascular function/flow-mediated dilation
sICAM-1/sVCAM-1/sP-Selectin	Soluble Intercellular/Vascular Cell Adhesion Molecules/Selectin P
EBC	Exhaled breath condensate
CRAE/CRVE	Central retinal arteriolar/venular equivalents
FBF	Forearm blood flow
AMC	Airway macrophage carbon
PRISMA	Preferred Reporting Items for Systematic Reviews and Meta-Analyses
PICO	Patient, Intervention, Comparison, Outcome (framework for research questions)
